# Parental depression and child outcomes – is marital conflict the missing link?

**DOI:** 10.1111/j.1365-2214.2011.01270.x

**Published:** 2012-07

**Authors:** L Hanington, J Heron, A Stein, P Ramchandani

**Affiliations:** * Department of Psychiatry, University of OxfordOxford; † Department of Social Medicine, University of BristolBristol, UK

**Keywords:** ALSPAC, marital conflict, maternal depression, paternal depression

## Abstract

**Background:**

Both maternal and paternal depression during the perinatal period are associated with adverse effects on child outcomes. Attention has started to focus on the mechanisms mediating these relationships. Marital conflict may play a role in this context.

**Methods:**

In a large cohort study, the Avon Longitudinal Study of Parents and Children (*n*= 14 541 pregnancies), we aimed to (i) investigate the relative influences of parental postnatal depression and marital conflict on child outcomes and to attempt to determine the pathway(s) of risk; (ii) investigate the impact of two types of antenatal stress (parental depression and marital conflict) on child outcomes; and (iii) determine the relative contributions of antenatal and postnatal risk. Parents completed the Edinburgh Postnatal Depression Scale and a marital conflict scale during the second trimester and at 8 months postnatally. Child outcomes were assessed at 42 months using the Rutter revised pre-school scales.

**Results:**

Marital conflict partially mediated the relationship between postnatal depression in both mothers and fathers and child outcomes, and acted as an independent risk for adverse outcomes. Parental depression (maternal and paternal) and marital conflict in the antenatal period were both associated with adverse effects which persisted even when postnatal stresses were taken into account.

**Conclusions:**

These findings, if replicated, suggest that screening and intervention programmes targeted at parental depression and marital problems should be considered antenatally, as well as postnatally.

## Introduction

The impact of postnatal depression on child development has received considerable attention. Children whose mothers are depressed are more likely to have cognitive, attachment and behavioural difficulties (Murray 1992; Murray *et al*. 1996a; Beardslee *et al*. 1998; Martins & Gaffan 2000). Physical development is also at risk (O'Brien *et al*. 2004). Similarly, children of depressed fathers are more likely to have adverse behavioural and emotional outcomes (Ramchandani *et al*. 2005, 2008), and adolescents have increased rates of psychopathology if their father is depressed (Kane & Garber 2004). In addition, it is clear that maternal depression in the antenatal period can have a lasting influence on the foetus. A large body of work supports the notion that if a mother is stressed during pregnancy (for example, due to natural disasters, depression or anxiety), her child is at increased risk of cognitive, emotional and behavioural difficulties (O'Connor *et al*. 2002; LaPlante *et al*. 2004; Niederhofer & Reiter 2004; Van den Bergh *et al*. 2005; Talge *et al*. 2007). These effects appear to continue through adolescence, with children of mothers who were depressed during the antenatal period being at increased risk for depression and antisocial outcomes (Pawlby *et al*. 2009; Hay *et al*. 2010). However, little is known about the effects of paternal depression in the antenatal period, and the relative contributions of antenatal and postnatal risks are yet to be determined.

Recently, research has started to focus on the mechanisms mediating the relationship between parental depression and adverse child outcomes. Four main pathways for the transmission of risk have been proposed, namely genetic heritage, impairment of neuroregulatory systems, the experience and effects (for example, on parenting) of the negative behaviours and cognitions associated with depression, and exposure to environmental stress (Goodman & Gotlib 1999). Families with a depressed mother report higher levels of stress in a number of domains (relationships, work, money, children) (Hammen *et al*. 1987), as well as a larger number of stressors (Hammen 1991), than families in which the mother is well. Marital conflict has been highlighted as a form of stress that deserves particular consideration in this context, as it has the potential to impact significantly on all family members. The association between marital conflict and depression has been recognized for many years (Briscoe & Smith 1973; Weissman & Paykel 1974). There are higher rates of both marital conflict (Johnson & Jacob 1997) and divorce (Coyne 1990) among depressed women, and a recent meta-analysis found that 18% of the variance in depressive symptoms among wives (14% among husbands) can be attributed to marital dissatisfaction (Whisman 2001).

The link between marital conflict and child behavioural, emotional and cognitive problems has been well established (Grych & Fincham 1990; Cummings & Davies 1994, 2002). Marital conflict may impact on children either directly or indirectly, for example, via disruptions in the parent–child relationship. Meta-analyses support the idea that problems in the marital relationship may ‘spillover’ into the parent–child relationship, leading to interaction styles that affect child outcomes (Erel & Burman 1995; Krishnakumar & Buehler 2000). However, other mechanisms must also be at work, as conflict to which children are exposed is more damaging than conflict which occurs when they are not present (Cummings & Davies 2002). The effects of marital conflict on child adjustment have been shown to be mediated by child emotional security (Davies *et al*. 2002; Cummings *et al*. 2006; Du Rocher Schudlich & Cummings 2007). Emotional security theory suggests that any factor, such as marital conflict, which threatens children's feelings of safety and security within the family, has the potential to affect well-being (Davies & Cummings 1994). The attributions that children, particularly older children, give to marital conflict may also be important in determining outcomes according to the cognitive–contextual framework (Grych & Fincham 1990), and these attributions may affect children's interpretation and expectations of conflict in their own interactions with their parents, thus impacting on child adjustment (Harold & Conger 1997).

Only a few studies have specifically investigated marital conflict as a mediator between parental depression and adverse child outcomes, and they tend to focus on older children and adolescents (Fergusson *et al*. 1995; Davies & Windle 1997; Du Rocher Schudlich & Cummings 2003; Weinfield *et al*. 2009). However, the results are fairly consistent in supporting the notion that marital conflict might explain at least part of the link between maternal depression and adverse child outcomes. The picture regarding paternal depression is less clear. For example, marital quality was found to play a significant mediational role in the association between paternal depressive symptomatology and child internalizing problems in a community sample of 235 parents of kindergarten children (Cummings *et al*. 2005), but earlier research looking at child adjustment failed to find such a relationship (Miller *et al*. 1993). A recent study investigating the effects of antenatal stress on cognitive and behavioural outcomes (Bergman *et al*. 2007) has reignited interest in the idea that marital conflict may have a significant and lasting impact on the unborn foetus (Stott 1973). Antenatal stress was found to account for 10% of the variance in child fearfulness at 14–19 months and 17% of the variance in cognitive ability, even after taking postnatal factors into account. Notably, 75% of the variance in child fearfulness that was related to antenatal stress could be accounted for by partner relationship strain in the antenatal period (the equivalent figure for cognitive ability was 73.5%).

The current study aims to test the hypothesis that marital conflict mediates the association between postnatal parental depression and adverse child outcomes. It also aims to add to existing knowledge by investigating the impact of two forms of antenatal stress (parental depression, including paternal depression, and marital conflict) on child behavioural and emotional outcomes, and attempting to disentangle the relative contributions of antenatal and postnatal risks (Thapar & Rutter 2009).

## Methods

### Participants

The Avon Longitudinal Study of Parents and Children (ALSPAC) (Golding *et al*. 2001) is a large longitudinal cohort study designed to collect a wide range of data on parents and their children from early pregnancy onwards. Pregnant women who were resident in Avon and had an expected delivery date between 1 April 1991 and 31 December 1992 were eligible to participate. The initial sample consisted of 14 541 pregnant women, with 14 676 foetuses. There were 14 062 live births and 13 988 children were alive at 1 year. Ethical approval for the study was obtained from the ALSPAC Law and Ethics Committee and the Local Research Ethics Committees.

A number of studies have been carried out to determine the representativeness of the sample used in the ALSPAC study as compared with the populations of both the Avon area and the UK as a whole. As has been found with other cohorts, there is a slight deficit of less affluent families and ethnic minorities in the ALSPAC sample (http://www.alspac.bristol.ac.uk). In total, 48.3% of children in the study were female; 97.4% of mothers and 96% of fathers were White, and 95% of the children were classed as White. Social class is occupationally defined in the UK, with five categories ranging from I (professional) to V (unskilled). The majority of parents in the ALSPAC sample (50.6% of mothers and 42.3% of fathers) fell into social class III (skilled); 11.0% of fathers and 5.9% of mothers were in social class I, with only 2.2% of mothers and 2.9% of fathers in social class V.

### Measures

Mothers and fathers completed the Edinburgh Postnatal Depression Scale (EPDS) at 18 weeks gestation (Time 1) and at 8 months postnatally (Time 2). The EPDS, a self-report questionnaire comprising 10 items, has been validated for use both during and outside the postnatal period in women, and also in men (Cox *et al*. 1987, 1996; Matthey *et al*. 2001). It is not a diagnostic tool, but scores of greater than 12 predict major depressive disorder with a high sensitivity (81.1%) and specificity (95.7%) (Murray & Carothers 1990) in women. In men the comparable sensitivity ranges are 71–86% and specificity 75–94% (Matthey *et al*. 2001; Ramchandani *et al*. 2008). In this study the EPDS data were split so that parents scoring greater than 12 were classed as ‘depressed’, while those scoring 12 or less were classed as ‘not depressed’.

Marital conflict was assessed using a nine-item scale which includes questions such as ‘Do you get angry with your partner?’ and ‘Does your partner listen when you want to talk about your feelings?’. Possible answers range from ‘almost always’ to ‘never’. The scale was developed for the purposes of the ALSPAC study, and has been used in other research (O'Connor *et al*. 2005b). Two composite scores, broadly entitled ‘Affection’ and ‘Aggression’, were initially calculated; however, preliminary analyses identified a single factor structure (Cronbach's α > 0.8 for maternal and the paternal data at each time point), and the scores were then recoded and merged to create an overall measure of marital conflict. Again, data were collected at 18 weeks gestation (Time 1) and at 8 months postnatally (Time 2). A top 10% cut-off was used to identify the most troubled relationships.

Child outcomes were assessed when the child was aged 42 months (Time 3) using the Rutter revised pre-school scales (Elander & Rutter 1996). Mothers were asked to give one of three responses (‘Yes certainly’, ‘Yes sometimes’ and ‘No’) to describe how accurately each of a number of statements regarding particular behaviours or characteristics related to their child. Examples of these statements include ‘Nowadays my child fights with other children’, and ‘Nowadays my child is worried, worries about many things’. Responses to individual statements were combined to create scores relating to emotional, conduct and total problems. Initially, analyses were carried out using the continuous data, and were then repeated with the data split using a top 10% cut-off to identify children with particular difficulties. This cut-off has been used in previous studies (Plomin *et al*. 2002; Ramchandani *et al*. 2005), and with other commonly used measures, such as the Strengths and Difficulties Questionnaire (Goodman & Scott 1999; Goodman 2001).

### Statistical analysis

1. Descriptive statistics were produced for all continuous variables, and the correlations between these variables were calculated. Second twins were excluded from all analyses to avoid familial clustering effects. All analyses were carried out using spss version 16.
2. Paired samples *t*-tests were carried out to compare maternal marital conflict scores at each time point to see whether there was any difference in mean score antenatally as compared with postnatally. The analyses were then repeated using the paternal marital conflict scores.
3. Simple regression analyses were conducted using the continuous data to examine any association between maternal depression and child outcome. Separate analyses were conducted for antenatal and postnatal depression. Initially, all analyses were carried out using total child problems as the outcome measure. They were then repeated using first emotional and then conduct difficulties as the outcome measures to see if any patterns emerged when emotional and behavioural problems were separated out. These analyses were then extended to include the following variables:
  - marital conflict (maternal or paternal rating) (the relationship between marital conflict and child outcomes was also assessed separately);
  - both maternal and paternal depression;
  - marital conflict (maternal or paternal rating) and maternal and paternal depression.
4. The analyses in step 3 were then repeated for the paternal depression data.
5. In order to establish and estimate the clinical relevance of any findings, and to enable calculation of odds ratios, the analyses set out in steps 3 and 4 were then repeated using the dichotomized variables described in the *Methods* section. Logistic regression was used for these analyses.
6. The above analyses were then repeated controlling for mothers' highest educational status.
7. The analyses described above (which follow the recommendations of Baron and Kenny (1986) for testing mediation) were then extended to include an assessment of the significance of the mediating effect of marital conflict (the indirect effect) using the bootstrapping method suggested by Preacher and Hayes (2004, 2008). The additional analyses were carried out to help increase statistical power and reduce the risk of error. An spss macro (‘Indirect’) was downloaded from http://www.afhayes.com/spss-sas-and-mplus-macros-and-code.html for the purpose. For each calculation the number of bootstrap re-samples was set at 5000.
8. Finally, all antenatal and all postnatal variables were included in a single model with each of the child outcome variables in turn to try and assess the relative importance of both antenatal and postnatal risks.

All results presented below are derived from analyses using maternal marital conflict ratings. The maternal conflict ratings were chosen as (i) the maternal and paternal conflict ratings were reasonably strongly correlated, and analyses revealed a broadly similar pattern of results for either measure, and (ii) maternal conflict data were available for a larger proportion of the study sample.

## Results

### Response rates

Overall, response rates were higher for maternal than paternal measures (see Table 1), and as expected, they decreased with time. Nonetheless, data were available on approximately 68% of mothers and 49% of fathers at Time 2, and 68% of children at Time 3, and sample sizes were large.

**Table 1:** Descriptive statistics

Variable	*n*	Min	Max	Mean	SD
Maternal conflict (antenatal)	11 725	9	45	19.40	5.21
Paternal conflict (antenatal)	8 338	9	41	19.12	5.01
Maternal conflict (postnatal)	10 390	13	64	29.65	8.44
Paternal conflict (postnatal)	6 914	13	62	29.18	8.10
Maternal depression (antenatal)	11 954	0	30	6.98	4.86
Maternal depression (postnatal)	9 846	0	27	4.23	3.93
Paternal depression (antenatal)	11 198	0	29	5.40	4.68
Paternal depression (postnatal)	7 090	0	24	3.35	3.70
Total child difficulties (42 months)	9 910	0	52	12.53	5.72
Conduct difficulties (42 months)	9 910	0	16	3.63	2.36
Emotional difficulties (42 months)	9 910	0	12	2.55	1.74

### Descriptive statistics

In total, 13.8% (1655/11 954) of mothers were classed as depressed in the antenatal period (Time 1), compared with 4% of fathers (395/9846). Of the 11 198 mothers who completed the EPDS in the postnatal period (Time 2), 980 (8.8%) were classed as depressed. Only 2.9% of fathers (209/7090) were classed as depressed at Time 2. Three hundred and sixty mothers and 59 fathers were depressed at both time points. The finding that rates of postnatal depression were higher among women than men is consistent with the results of previous studies.

Overall, marital conflict scores were similar for mothers and fathers. Both mothers (mean = 29.59, SD = 8.37) and fathers (mean = 28.98, SD = 7.93) reported higher levels of marital conflict after the birth of their child than they did antenatally [maternal conflict mean = 19.22, SD = 5.08, *t*(9757) =−164.41, *P* < 0.001; paternal conflict mean = 18.84, SD = 4.91, *t*(5972) =−129.27, *P* < 0.001]. A proportion of 46.5% of mothers who reported high levels of marital conflict (i.e. in the top 10%) at Time 1 were still reporting high levels of conflict at Time 2.

Correlations between all variables were statistically significant at *P* < 0.001 (see Table 2).

**Table 2:** Correlation matrix

	Matdep1	Patdep1	Matdep2	Patdep2	Matcon1	Patcon1	Matcon2	Patcon2	Emotion	Conduct	Total
Matdep1	1										
Patdep1	0.25	1									
Matdep2	0.50	0.17	1								
Patdep2	0.22	0.56	0.28	1							
Matcon1	0.28	0.17	0.24	0.15	1						
Patcon1	0.20	0.25	0.15	0.21	0.65	1					
Matcon2	0.28	0.19	0.38	0.24	0.67	0.51	1				
Patcon2	0.18	0.26	0.24	0.37	0.48	0.64	0.64	1			
Emotion	0.18	0.08	0.18	0.07	0.09	0.05	0.11	0.07	1		
Conduct	0.15	0.07	0.18	0.07	0.16	0.11	0.17	0.10	0.24	1	
Total	0.22	0.11	0.25	0.11	0.16	0.10	0.19	0.12	0.64	0.76	1

All of the above correlations are significant at *P* < 0.001.

Matdep1, maternal depression (antenatal); Patdep1, paternal depression (antenatal); Matdep2, maternal depression (postnatal); Patdep2, paternal depression (postnatal); Matcon1, antenatal marital conflict (maternal score); Patcon1, antenatal marital conflict (paternal score); Matcon2, postnatal marital conflict (maternal score); Patcon2, postnatal marital conflict (paternal score).

### Postnatal depression as a predictor of child outcome

There was strong evidence found that both postnatal maternal (OR 2.79, 95% CI 2.30–3.40) and postnatal paternal (OR 2.20, 95% CI 1.47–3.28) depression predicted total child problems at age 42 months (see Table 3). When postnatal marital conflict scores were included, the association between depression and total problems was attenuated by approximately 25% but remained substantial for mothers (OR 2.34, 95% CI 1.88–2.91). Similarly, inclusion of marital conflict attenuated the relationship for fathers by approximately 17.6%, but the evidence for an independent effect of paternal depression remained strong (OR 1.98, 95% CI 1.31–2.99). Bootstrapping analyses revealed the indirect effect of marital conflict to be significant (see Table 4). Marital conflict partially mediates the relationship between parental depression and child outcomes, and is also an independent predictor of adverse child outcomes itself (see Table 3).

**Table 3:** Depression in the postnatal period and child outcome (logistic regression analyses)

	Number of children	% children with poor outcome	Unadjusted OR (95% CI)	OR adjusted for marital conflict (maternal rating) (95% CI)	OR adjusted for depression in other parent	OR adjusted for marital conflict (maternal rating) and depression in other parent
A. Total child problems						
Mother not depressed	8598	7.92	2.79** (2.30–3.40)	2.34** (1.88–2.91)	2.62** (2.02–3.39)	2.31** (1.76–3.02)
Mother depressed	769	19.38				
Father not depressed	6036	8.35	2.20** (1.47–3.28)	1.98* (1.31–2.99)	1.79* (1.18–2.71)	1.71* (1.13–2.61)
Father depressed	180	16.67				
B. Conduct difficulties						
Mother not depressed	8598	10.50	2.29** (1.90–2.76)	1.87** (1.52–2.30)	1.99** (1.54–2.57)	1.74** (1.33–2.52)
Mother depressed	769	21.20				
Father not depressed	6036	10.22	1.83* (1.23–2.72)	1.68* (1.12–2.52)	1.59* (1.06–2.38)	1.53* (1.02–2.30)
Father depressed	180	17.22				
C. Emotional difficulties						
Mother not depressed	8598	12.20	1.86** (1.54–2.24)	1.74** (1.42–2.14)	1.78** (1.39–2.27)	1.75** (1.36–2.52)
Mother depressed	769	20.55				
Father not depressed	6036	12.77	1.31 (0.88–1.97)	1.31 (0.87–1.97)	1.17 (0.78–1.77)	1.19 (0.78–1.80)
Father depressed	180	16.11				

* *P* < 0.05;

** *P* < 0.001.

**Table 4:** Bootstrap results of indirect effects

Independent variable	Mediator	Dependent variable	Point estimate	Bias	Standard error	95% CI (bias corrected and accelerated)
Antenatal maternal depression	Antenatal marital conflict (maternal score)	Total	0.0388	0.0001	0.0042	0.0307–0.0473
Antenatal maternal depression	Antenatal marital conflict (maternal score)	Conduct	0.0197	0.0000	0.0018	0.0162–0.0233
Antenatal maternal depression	Antenatal marital conflict (maternal score)	Emotional	0.0041	0.0000	0.0012	0.0018–0.0065
Antenatal paternal depression	Antenatal marital conflict (maternal score)	Total	0.0369	0.0000	0.0040	0.0297–0.0454
Antenatal paternal depression	Antenatal marital conflict (maternal score)	Conduct	0.0155	0.0000	0.0017	0.0125–0.0191
Antenatal paternal depression	Antenatal marital conflict (maternal score)	Emotional	0.0058	0.0000	0.0010	0.0039–0.0078
Postnatal maternal depression	Postnatal marital conflict (maternal score)	Total	0.0543	−0.0001	0.0057	0.0431–0.0655
Postnatal maternal depression	Postnatal marital conflict (maternal score)	Conduct	0.0231	0.0000	0.0024	0.0185–0.0279
Postnatal maternal depression	Postnatal marital conflict (maternal score)	Emotional	0.0067	0.0000	0.0017	0.0035–0.0101
Postnatal paternal depression	Postnatal marital conflict (maternal score)	Total	0.0644	0.0000	0.0060	0.0532–0.0768
Postnatal paternal depression	Postnatal marital conflict (maternal score)	Conduct	0.0229	0.0000	0.0024	0.0186–0.0280

The results were broadly similar when looking at conduct difficulties. However, when emotional difficulties was used as the outcome measure, paternal depression was found not to be a significant predictor of child outcome.

### Antenatal depression as a predictor of child outcome

Antenatal maternal (OR 2.43, 95% CI 2.03–2.91) and paternal (OR 2.34, 95% CI 1.70–3.23) depression each predicted later total problems. When antenatal marital conflict was added to the models, the association between depression and child total problems decreased slightly (maternal depression OR 2.16, 95% CI 1.78–2.63; paternal depression OR 2.17, 95% CI 1.54–3.05). Nonetheless, both maternal and paternal depression and marital conflict remained strong independent predictors of total problems at 42 months (see Table 5). Again, bootstrapping analyses revealed the indirect effect of marital conflict to be significant (see Table 4).

**Table 5:** Depression in the antenatal period and child outcome (logistic regression analyses)

	Number of children	% children with poor outcome	Unadjusted OR (95% CI)	OR adjusted for marital conflict (maternal rating) (95% CI)	OR adjusted for depression in other parent	OR adjusted for marital conflict (maternal rating) and depression in other parent
A. Total problems						
Mother not depressed	7893	7.73	2.43** (2.03–2.91)	2.16** (1.78–2.63)	2.29** (1.86–2.83)	2.08** (1.67–2.60)
Mother depressed	1065	16.90				
Father not depressed	7382	8.57	2.34** (1.70–3.23)	2.17** (1.54–3.05)	1.88** (1.32–2.66)	1.89* (1.31–2.71)
Father depressed	272	18.01				
B. Conduct difficulties						
Mother not depressed	7893	10.39	2.02** (1.70–2.39)	1.74** (1.45–2.09)	2.01** (1.64–2.45)	1.80** (1.46–2.22)
Mother depressed	1065	18.97				
Father not depressed	7382	10.78	1.47* (1.05–2.06)	1.28 (0.89–1.85)	1.19 (0.82–1.73)	1.10 (0.74–1.62)
Father depressed	272	15.07				
C. Emotional difficulties						
Mother not depressed	7893	11.73	2.07** (1.76–2.44)	2.00** (1.68–2.38)	1.95** (1.61–2.35)	1.89** (1.56–2.30)
Mother depressed	1065	21.60				
Father not depressed	7382	12.86	1.68** (1.24–2.28)	1.56* (1.12–2.16)	1.46* (1.05–2.03)	1.39 (0.98–1.97)
Father depressed	272	19.85				

* *P* < 0.05;

** *P* < 0.001.

There were some differences in the results obtained when emotional and conduct difficulties were used separately as the outcome measures. Once more, antenatal maternal depression and antenatal paternal depression each individually predicted later conduct difficulties, but paternal depression did not when antenatal conflict was included in the model. The findings were similar for emotional difficulties, although in this case marital conflict also did not predict child emotional problems in the overall model (see Table 5).

### Overall models

When all variables were included in a single model, all except postnatal paternal depression were found to be important predictors of later total problems. Antenatal risks appear, broadly, to be as important as postnatal risks in determining child outcomes. When examining conduct and emotional difficulties separately, paternal depression did not predict conduct difficulties and marital conflict was only significant in the antenatal period. Only antenatal and postnatal maternal depression predicted emotional outcomes.

Overall, similar results were obtained when maternal highest educational qualification was added as a potential confounder.

## Discussion

The main findings of our study can be summarized as follows:

1. Parental depression in the postnatal period was associated with adverse child outcomes. More specifically, maternal postnatal depression significantly predicted later child total, conduct and emotional outcomes, with marital conflict partially mediating these relationships as well as contributing unique variance. Postnatal paternal depression was found to predict later total problems and conduct difficulties in children, and marital conflict again partially mediated these relationships.
2. In line with the existing literature, our results suggest that antenatal stress is associated with negative effects which remain apparent several years after birth and which are significant even when postnatal stresses are taken into account. Maternal depression in the antenatal period was independently linked with emotional, conduct and total problems in the child later on. Both paternal depression and marital conflict in the antenatal period were associated with higher total problem scores at 42 months, and antenatal conflict also predicted later conduct difficulties.
3. Finally, marital conflict scores were found to increase by more than 50% between the antenatal and postnatal periods.

This study has a number of strengths. The sample size was very large, limiting selection bias. The longitudinal design allowed us to test for postnatal confounds of antenatal stress. Furthermore, the EPDS and Rutter scales have been widely used and well validated.

Several limitations must be considered. First, as is often the case in such studies, response rates were lower for fathers than for mothers, giving rise to the possibility of response bias. For example, the impact of paternal depression on child outcomes may have been underestimated if depressed fathers were less likely to take part in the study. Second, there may have been an element of rater bias as maternal report measures were used to assess child outcomes, and depressed mothers might rate their children as having more problems. This could also have led to an overestimation of maternal, as compared with paternal, effects.

The findings of this study suggest that marital conflict only partially mediates the relationship between postnatal parental depression and behavioural and emotional outcomes in children, suggesting that other pathways of risk also exist. Parenting may represent one such pathway. Mothers who are depressed have been shown to display decreased sensitivity and responsiveness towards their offspring, and tend to be more punitive and less positive in their parenting (Murray & Cooper 2003). Such interaction styles have been associated with adverse cognitive outcomes in their children (Murray *et al*. 1996a, b). Individual child characteristics also deserve consideration. For example, the role of gender remains unclear. In the context of this study, it is also worth noting that using an overall measure of marital conflict may have led to its role as a mediator being underestimated. It has been suggested that the way in which conflict is dealt with is more important than, for example, the frequency with which it occurs (Goodman *et al*. 1999). The use of constructive conflict tactics (problem solving, displaying affection, etc.) can have positive effects on children, helping them to develop effective ways of problem solving and dealing with conflict (Grych & Fincham 1990) and reducing the chance of behaviour problems (Cummings *et al*. 2004). Conversely, both destructive (aggression, the use of insults, etc.) and depressive (for example, withdrawal) tactics have been linked with adverse child outcomes (Du Rocher Schudlich & Cummings 2003, 2007). It may be that a conflict measure focusing on destructive or depressive tactics (and excluding constructive ones) may have led to conflict playing a stronger meditational role.

The finding that antenatal parental depression and marital conflict are both significant predictors of child outcomes even after postnatal variables have been taken into account lends support to the programming hypothesis, which proposes that the antenatal environment can have long-lasting effects on offspring development, the consequences of which may persist into adulthood (Weinstock 1996; Gluckman *et al*. 2005; Levine 2005; O'Connor *et al*. 2005a). Several potential mechanisms of transmission deserve consideration. For example, it may be that if the mother experiences stressful events during pregnancy, the foetus is exposed to higher levels of stress hormones, leading to risks for the child (Talge *et al*. 2007). Second, epigenetic factors may also play a role (Zhang *et al*. 2004; Kappeler & Meaney 2010). Alternatively, adverse aspects of the environment may both act as stressors for the mother and impact negatively on the offspring during early postnatal life. Genetic factors are also likely to be important. Further work is necessary to determine the nature of the antenatal stresses that are particularly likely to raise the risks for the unborn child and whether a particular sensitive period exists. Studies designed to answer these questions would prove invaluable in directing interventions to prevent damage at vulnerable times. It is worth commenting that marital conflict may be associated with an increased risk of domestic violence, and pregnant women are an at-risk group in this respect. The findings of Bergman and colleagues (2007) suggest that domestic violence has the potential to impact on the unborn child, highlighting this issue as a public health concern.

Our finding that marital conflict scores increase significantly after the birth of the child is consistent with the results of previous studies. Parents tend to report lower levels of satisfaction in their relationships than non-parents (Twenge *et al*. 2003) and it has been shown that there is a sudden decrease in marital quality following the birth of a child (Doss *et al*. 2009). There are several explanations for these findings. Marital satisfaction has been shown to decrease over time, particularly during the first years of marriage (Karney & Bradbury 1997; Huston *et al*. 2001). Additionally, parents may experience a period of enhanced cooperation during pregnancy, and the observed increase in conflict may simply be due to a return to pre-pregnancy levels (Feeney *et al*. 2001; Huston & Holmes 2004). Having a child is associated with a number of stressors, and these may be a source of conflict. Parents also have greater demands on their time and less opportunity to focus on their relationships. It is interesting that the observed increase in marital conflict occurs in the context of decreased depressive symptoms in both mothers and fathers.

## Conclusion

These findings highlight the potential importance of recognizing and treating both maternal and paternal depression so that child outcomes can be optimized. If these findings are replicated in other studies, screening programmes covering the antenatal and postnatal periods should be considered for both parental depression and marital conflict. In addition, appropriate interventions to enhance the couple relationship should be made more easily available.

**Key messages**

- Parental depression (both maternal and paternal) in the postnatal period is associated with adverse child outcomes.
- Marital conflict partially mediates the relationship between postnatal depression and child outcomes.
- Several different forms of antenatal stress (maternal depression, paternal depression and marital conflict) are associated with adverse child outcomes.
- Screening and intervention programmes targeted at parental depression and marital conflict may prove beneficial for child development.
